# Thermotolerant insect-associated selective pressure may drive increased lipid metabolic plasticity and emerging pathogenic potential in yeasts

**DOI:** 10.3389/fmicb.2026.1745440

**Published:** 2026-01-22

**Authors:** Casey Van Baalen, Nerve Zhou, Teun Boekhout, Olihile M. Sebolai, Jacobus Albertyn, Carolina H. Pohl

**Affiliations:** 1 Department of Microbiology and Biochemistry, University of the Free State, Bloemfontein, South Africa; 2 Department of Biological Sciences and Biotechnology, Botswana International University of Science and Technology, Palapye, Botswana; 3 College of Sciences, King Saud University, Riyad, Saudi Arabia; 4 The Yeasts Foundation, Amsterdam, Netherlands

**Keywords:** emerging pathogenic yeasts, fungal adaptation, insect-associated yeasts, lipid metabolism, One Health

## Abstract

The emergence of pathogenic yeasts such as *Candidozyma auris* represents a growing global health threat. Despite advances in fungal genomics, the ecological and physiological origins of pathogenicity in yeasts remain poorly understood. Most yeasts thrive at mesophilic temperatures, with a sharp decline in biodiversity beyond 30 °C, limiting the ability of many yeasts to infect endothermic hosts. Most insects, as ectothermic organisms with variable and often elevated body temperatures, co-exist intimately with yeasts in diverse environments and exert unique selective pressures, particularly regarding thermal stress. We hypothesise that these interactions potentially select for yeasts with enhanced lipid metabolic plasticity, a key trait underlying thermotolerance, immune evasion, nutrient adaptation, and antifungal resistance—attributes central to fungal virulence and pathogenicity and that thermotolerant insects thus act as ecological bridge for yeasts to move between the environment and endothermic hosts.

## 1 Introduction

**Figure 1:**
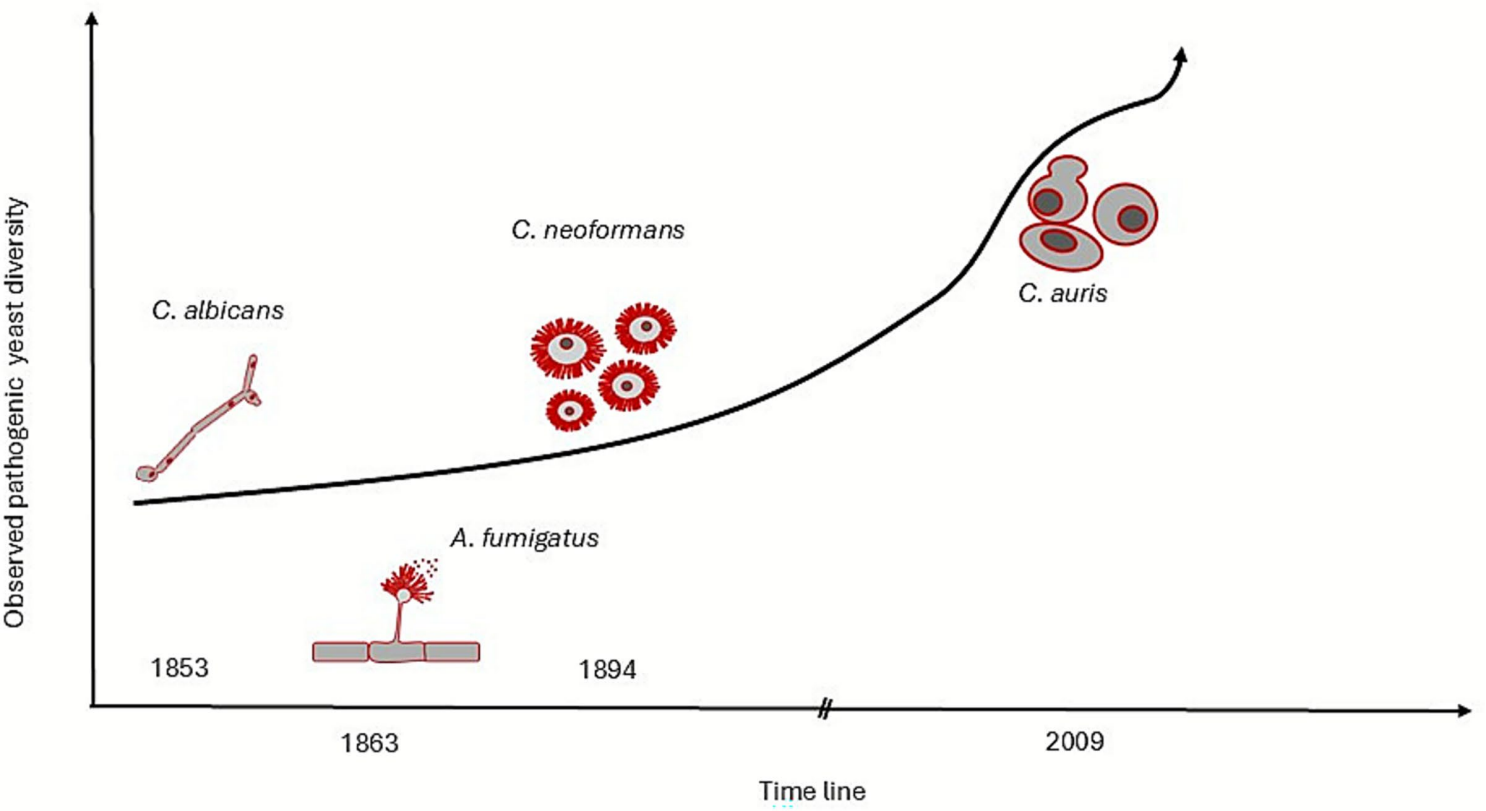
Increasing diversity of pathogenic yeasts over the past 200 years. The graph illustrates the growing number of identified pathogenic yeasts, including those within the critical fungal priority group (*Candida albicans*, *Aspergillus fumigatus*, *Cryptococcus neoformans*, and *Candidozyma auris*), reflecting advancements in identification techniques and the emergence of new species.

Of the millions of fungal species estimated to exist, only a small proportion consisting primarily of yeasts, has been documented as pathogenic to humans (Hazen, 1995; Perfect and Schell, 1996). Recent advances in identification techniques, coupled with ongoing exploration of fungal biodiversity, have led to the rapid characterisation of novel yeast species, revealing a concerning rise in emerging pathogenic yeasts (Boekhout et al., 2022) (Figure 1). In response, the World Health Organisation (WHO) addressed this issue by identifying and prioritising a list of fungal pathogens that require urgent research and public attention (World Health Organisation, 2022). However, the continued emergence of new fungal pathogens (Friedman and Schwartz, 2019; Uhrlaß et al., 2022; Oliveira et al., 2025) poses challenges, not only for identification, prioritisation, but also for surveillance and detection of novel fungal threats to human health.

The emergence of novel pathogenic yeasts, such as *Candidozyma* (*Candida*) *auris,* a nosocomial fungal pathogen with an elusive environmental origin, has challenged our understanding of the characteristics and drivers involved in their pathogenicity. Currently, most yeasts are adapted to mesophilic temperatures, exhibiting optimal growth between 12 °C and 30 °C, and for every 1 °C increase in temperature between 30 °C and 42 °C, 6% fewer strains can grow, suggesting a linear relationship between temperature and fungal growth tolerance (Robert and Casadevall, 2009). This thermal physiology constraint prevents many fungi, with latent pathogenic potential, from infecting humans (Neabore, 2024). However, a factor implicated in the emergence of novel human pathogenic yeasts is the influence of increased environmental temperature, which may facilitate their ability to breach the thermal barrier of endotherms (Robert et al., 2015; Centres for Disease Control, 2024; Ibe and Pohl, 2025).

Yeasts are globally distributed, yet their natural ecosystems remain poorly understood. Notably, these microorganisms commonly coexist with invertebrates, including insects, in diverse environmental settings, such as soil, water, and plant ecosystems, where their interactions influence each other’s ecology and evolution (Valenti et al., 2024). Vectoring and protection are among the benefits that yeasts gain from insects (Becher et al., 2018; Stefanini, 2018; Makopa et al., 2024). However, unlike endotherms, ectotherms (such as insects) are unable to regulate their body temperature and depend on external temperature, which directly affects their physiological processes and thereby their fitness (Deutsch et al., 2008; Kingsolver et al., 2013). The impact of rising global environmental temperatures may exert selective pressure on insect-associated yeasts, favouring thermotolerant strains capable of eventually breaching the thermal barrier of endotherms (Robert et al., 2015). In this context, thermotolerant insects may serve as ecological “thermal incubators,” exposing yeasts to fluctuating (i.e., diurnal and seasonal shifts in temperature) and elevated temperatures that pre-adapt them for survival in mammalian hosts (Caspeta and Nielsen, 2015; Caspeta et al., 2016; Garcia-Solache et al., 2013).

Although thermotolerance is an important characteristic of mammalian fungal pathogens, it is not sufficient to confer pathogenicity. Other characteristics, including the ability to withstand or evade other host-associated stresses such as immune responses and nutrient limitation, must also be present. In addition, for pathogenic yeasts to have human pandemic potential, they should be capable of efficient transmission to susceptible hosts and exhibit resistance to antifungal drug treatments (Jafarlou, 2024). However, despite these known requirements, no single underlying ability has been identified that universally allows for the transition from saprobic to parasitic lifestyles in yeasts, suggestive of a multifactorial and context-dependent phenomenon (Garcia-Solache et al., 2013).

Here, we hypothesise that inherent lipid metabolism plasticity, selected for by ectotherms with high preferred body temperature, such as thermotolerant insects, is a crucial characteristic. This ability informs the adaptability of yeasts to move between different niches, including mammals as potential pathogens. Thus, insect hosts may function as evolutionary incubators, wherein fluctuations in thermal and immune pressures drive the development of lipid plasticity, thereby pre-adapting yeasts for colonisation and survival in mammalian hosts.

## 2 Lipid metabolism as key to fungal adaptability

Historically considered difficult to analyse and of little importance beyond energy storage and membrane structure, lipids have not received the level of research attention of DNA, RNA and proteins in understanding cellular function and processes. However, recent technological advances in the field, particularly the advent of lipidomics, have indicated that lipid metabolism is fundamental to the regulation of many cellular processes (Santos and Preta, 2018).

Lipids are organised into eight major classes: free fatty acids, glycerolipids, glycerophospholipids, sterols, sphingolipids, prenol lipids, glycolipids, and polyketides (Klug and Daum, 2014). The lipidome of eukaryotic cells consists of hundreds of distinct lipid species produced by an integrated metabolic network involving many of these lipid classes (Klose et al., 2012). Cells adapt to environmental conditions by remodelling these complex lipidomes. This not only influences cellular architecture, but also other metabolic pathways which are required for physiological adaptations (Casanovas et al., 2015). As a result, variations in lipid profiles can serve as biomarkers of phenotypic adaptation to specific environmental conditions (Bianchi et al., 2020). Given this link between lipid plasticity and environmental adaptation, it is plausible that yeasts with specific lipid profiles may be more likely to emerge as pathogens in mammalian hosts.

**Figure 2:**
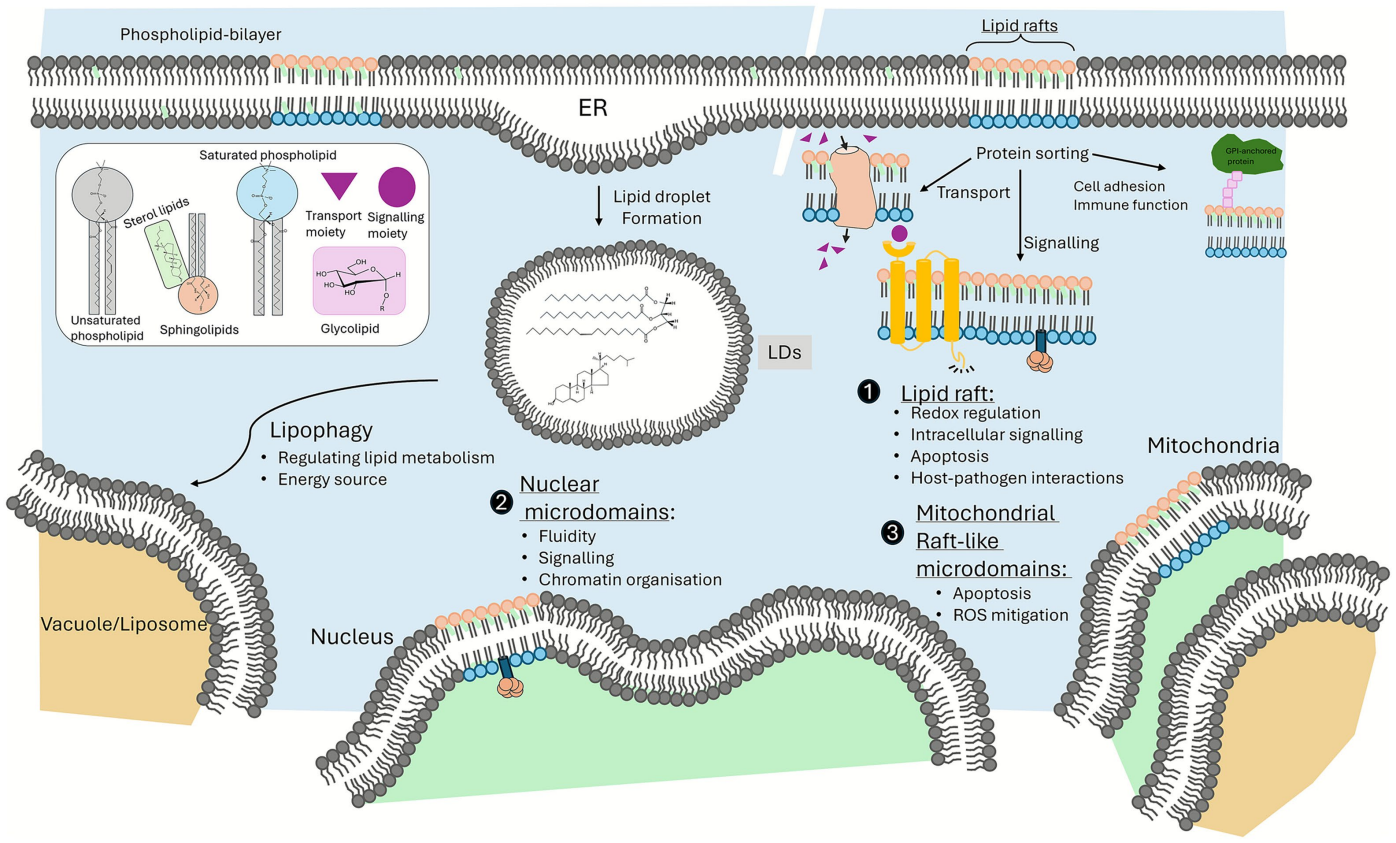
Lipid organisation and function in fungal membranes. The lipid bilayer is composed of unsaturated and saturated phospholipids, organised into distinct microdomains, including lipid rafts, mitochondrial raft-like microdomains, and nuclear microdomains. These microdomains perform various functions, such as redox regulation, intracellular signalling, apoptosis, host-pathogen interactions, ROS mitigation, membrane fluidity, signalling, and chromatin organisation. The formation of lipid droplets (LDs) from the endoplasmic reticulum (ER) is also depicted, illustrating their role in regulating lipid metabolism and serving as an energy source.

Lipid droplets (LDs) are highly dynamic organelles ubiquitous in eukaryotic cells that undergo cycles of growth and consumption, storing neutral lipids such as triglycerides and sterol esters within a phospholipid monolayer (Olzmann and Carvalho, 2019) (Figure 2). Notably, LDs provide multifaceted protection by buffering against endoplasmic reticulum stress, mitigating mitochondrial damage during autophagy, and serving as a lipid reserve, allowing for membrane expansion during cell growth under nutrient-poor conditions (Olzmann and Carvalho, 2019). Additionally, LDs also take part in regulating lipid metabolism, cellular metabolism, signalling and innate immunity in eukaryotic cells (Bosch et al., 2021; Brink et al., 2021) (Figure 2).

It is well known that lipid metabolism plays a central role in the ability of entomopathogenic fungi to colonise and infect insect hosts and that certain mechanisms (e.g., secretion of lipases) are common to both insect and mammalian pathogenic fungi (Keyhani, 2018). Interestingly, in this group of fungi, LDs and their proteins can participate in all stages of infection, from penetration to immune evasion to eventual sporulation. Beyond the role of LDs in direct virulence, and as an example of the importance of LDs in fungal adaptation, a recent study revealed that *C. auris* forms giant lipid droplets (gLDs) as a lipid-driven adaptive survival strategy in nutrient-poor conditions in the ear canals of mice (Zheng et al., 2025). gLDs were associated with distinct phenotypic changes, such as cell wall remodelling characterised by a thicker inner cell wall enriched with chitin. These cells exhibited enhanced thermotolerance at 42 °C and increased resistance towards human antimicrobial peptides. gLD-containing cells also showed increased fungal burden in multiple mouse organs and increased skin colonisation, demonstrating the extent to which lipids can influence the pathogenicity of yeasts.

In addition to the adaptive role of LDs, changes in membrane lipidomes modifies membrane properties such as viscosity, lipid packing, thickness, and phase properties (Chwastek et al., 2020; Lam et al., 2014) enabling tolerance towards temperature, osmotic stress, detergent exposure, nutrient limitations, and changes in cell densities. Beyond biophysical changes, various lipid classes also shape cell signalling that facilitates adaptive responses to environmental challenges (Figure 2). Lipids within cellular, mitochondrial, and nuclear membranes are organised into microdomains, primarily composed of phospholipids, sterols and sphingolipids, which organise signalling proteins (Wachtler and Balasubramanian, 2006). These lipid raft-like microdomains are essential for signalling pathways, protein sorting and function, as well as cellular homeostasis (Santos and Preta, 2018; Klug and Daum, 2014; Wachtler and Balasubramanian, 2006). In addition, fatty acids can post-translationally modify proteins (Wright et al., 2010; Nichols et al., 2015). The metabolism of sphingolipids also generates bioactive intermediates, such as ceramides, that act directly on signalling molecules, regulating stress responses (Klug and Daum, 2014). These findings highlight that the plasticity of fungal lipid metabolism is a critical mechanism that confers flexibility to pathogenic fungi to transition and adapt to many different conditions, including different environmental and host niches (Klose et al., 2012; Prasad and Singh, 2013).

## 3 Lipids and yeast virulence

Research has demonstrated the importance of lipids, including membrane lipids (Rella et al., 2016), and signalling lipids (Shea and Del Poeta, 2006) in the virulence of pathogenic yeasts (Mishra and Prasad, 1990; Mishra et al., 1992). Today, lipids are known to be integral to most aspects of fungal virulence, including survival within hosts as well as the expression of virulence factors and the development of drug resistance.

**Figure 3:**
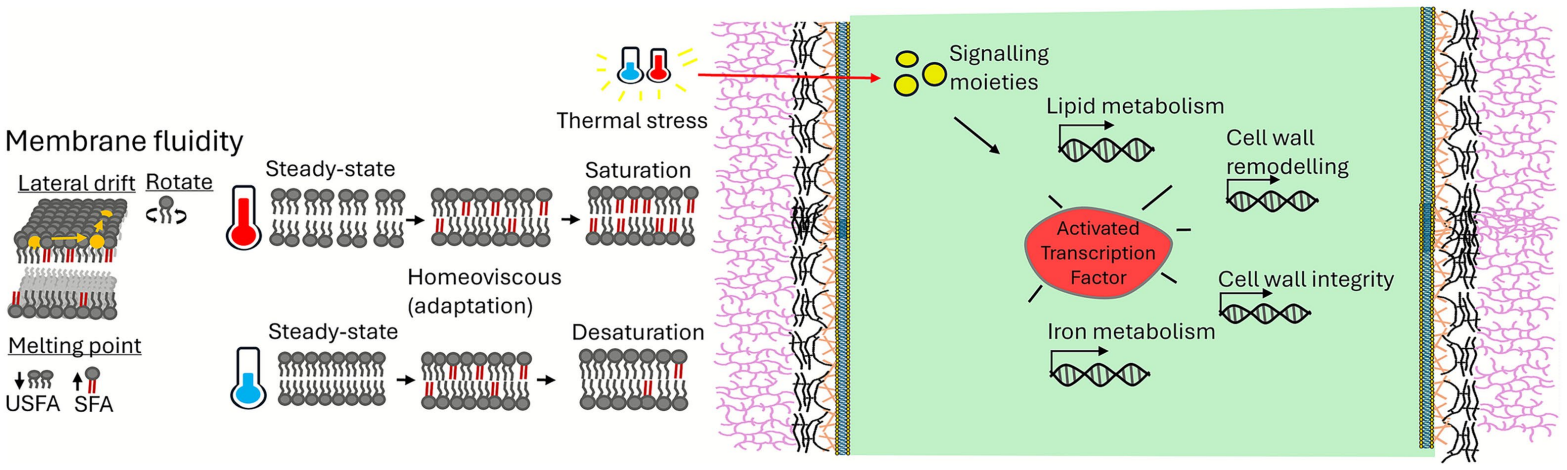
Thermal response in fungi. The illustration depicts how temperature affects membrane fluidity, highlighting the difference in melting points of saturated and unsaturated fatty acids. Temperature stress triggers a cascade of reactions, mediated by signalling molecules, which modulate genes involved in lipid metabolism, cell wall remodeling, cell wall integrity, and iron metabolism.

Thermotolerance is an essential factor enabling fungi to transition from a saprobic to a pathogenic lifestyle in mammalian hosts (Robert and Casadevall, 2009). Temperature stress triggers extensive lipid remodelling, including modifications in fatty acid desaturation levels and phospholipid composition, which helps to maintain membrane fluidity and mitigate oxidative stress damage (Fabri et al., 2023; Guerzoni et al., 1997; Ianutsevich et al., 2016) (Figure 3). Moreover, alterations in sphingolipid composition may activate intracellular signalling pathways, serving as a temperature-sensing mechanism in fungi (Xiao et al., 2022) (Figure 3).

**Figure 4:**
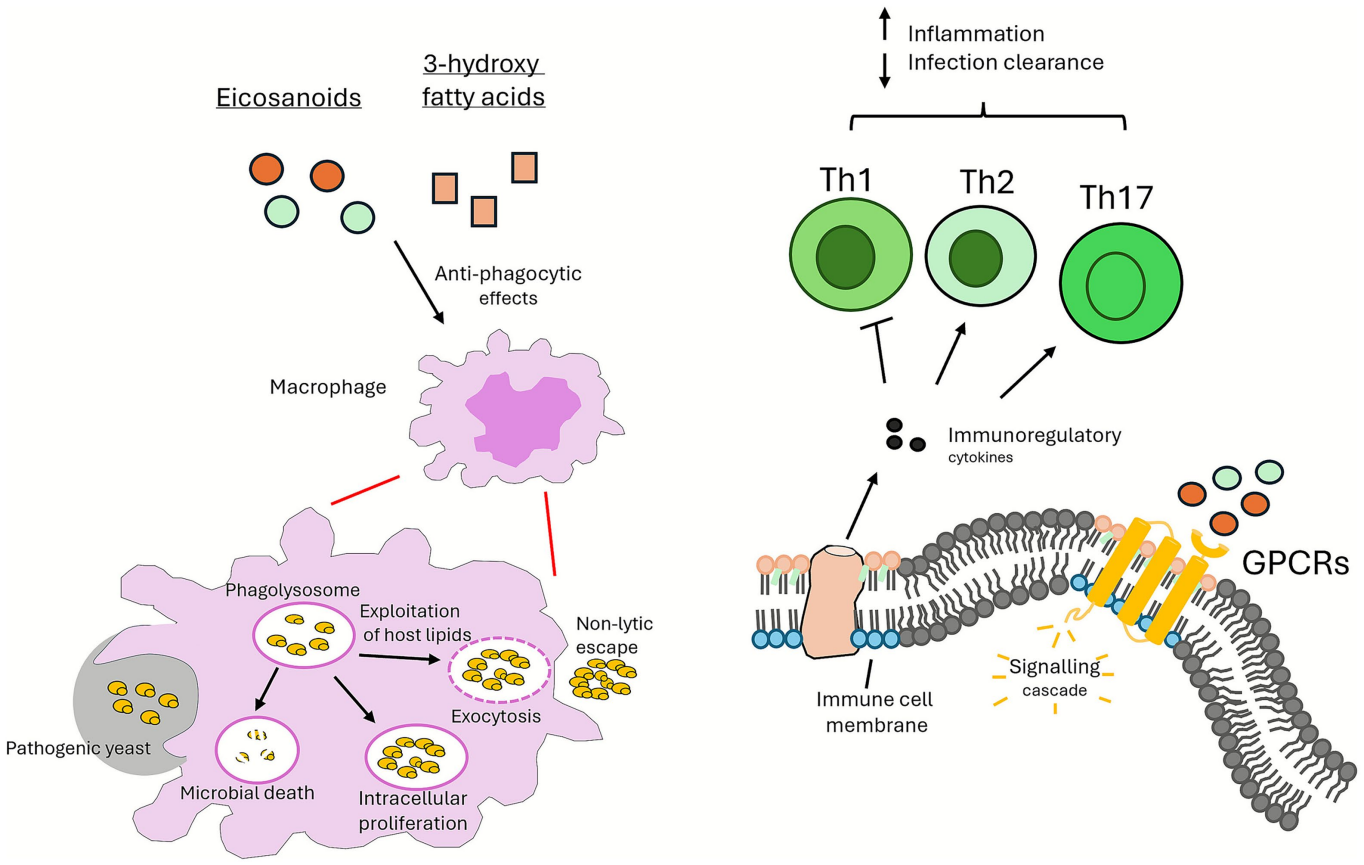
Lipid-mediated immune evasion strategies. Employed by pathogenic yeasts include the production of 3-hydroxy fatty acids, the exploitation of host-derived lipids, the modulation of host immune responses by fatty acid metabolites, and prostaglandin E_2_, enhancing survival and pathogenicity within the host.

Lipids are also central to immune evasion by fungi. In the basidiomycetous human pathogenic yeast, *Cryptococcus neoformans*, diacylglycerol regulates the expression of antiphagocytic protein (App1) and the activation of melanin, enhancing the yeast’s ability to evade host immune responses (Heung et al., 2005; Luberto et al., 2003; Mare et al., 2005). In addition, this yeast exploits host-derived lipids, such as macrophage-derived oleic acid, promoting replication and non-lytic escape from macrophages (Ells et al., 2012) (Figure 4). Oxidised lipids also play important immunomodulatory roles. *C. neoformans* secretes 3-hydroxy fatty acids, which protect the yeast from phagocytosis (Madu et al., 2015; Madu et al., 2017; Sebolai et al., 2007) (Figure 4), and the eicosanoids, prostaglandin E_2_ (PGE_2_) and resolvin E_1_, protect other human pathogenic yeasts, *Candida albicans* and *Candida parapsilosis*, from macrophage attack by modulating the host immune response (Ells et al., 2012; Haas-Stapleton et al., 2007; Tan et al., 2019).

**Figure 5:**
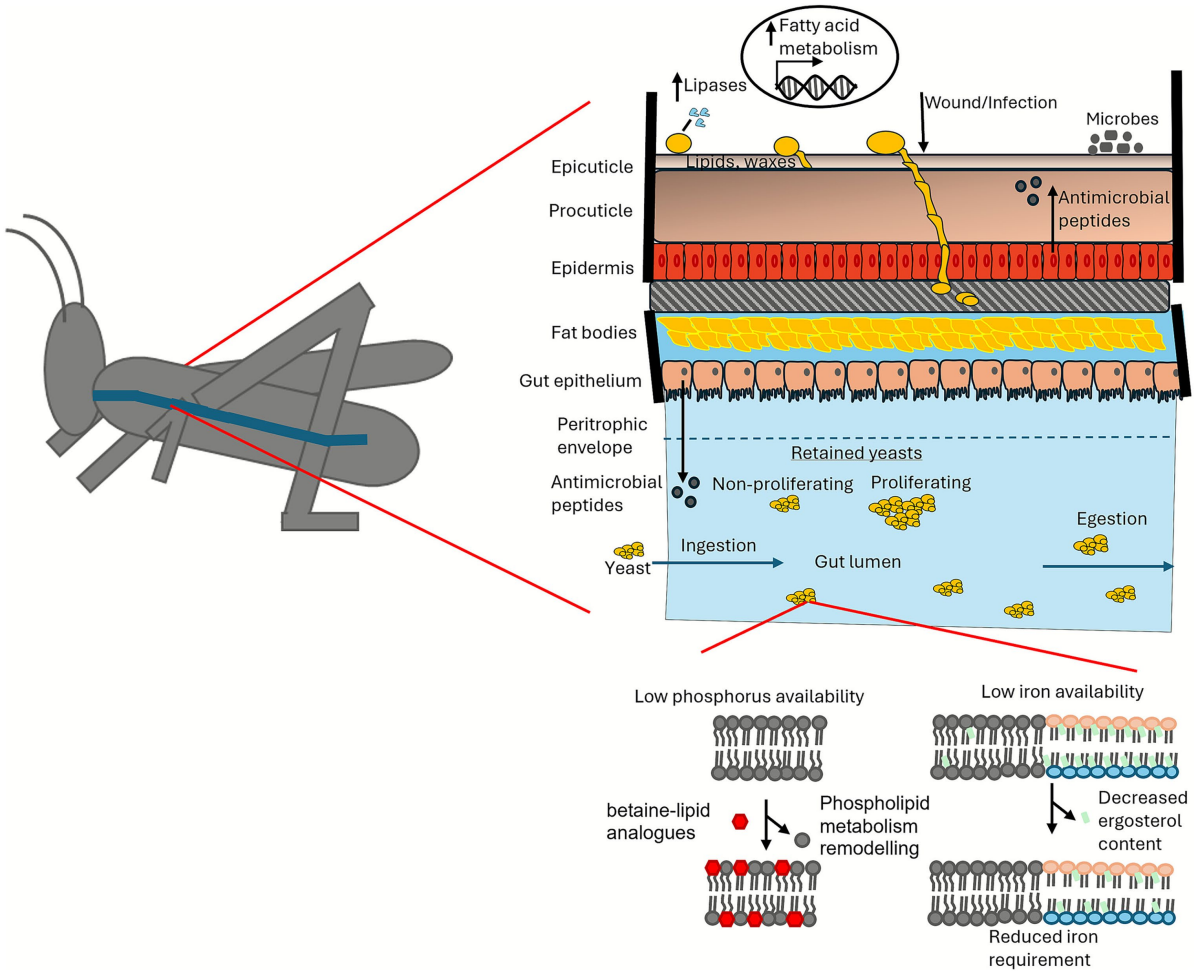
Potential adaptation of pathogenic fungi to nutrient-poor host conditions. The diagram illustrates how fungi utilise lipids and waxes from the surface to colonise the host. In addition, upon ingestion, yeasts are divided into proliferating and non-proliferating populations that are retained in the intestinal tract, while non-retained yeasts are egested. Additionally, the figure highlights the potential adaptive mechanisms of yeasts, including the replacement of phospholipids with betaine-lipid analogues to release phosphorus under low phosphorus conditions, and the reduction of ergosterol content to decrease iron requirements under low iron availability.

Nutrient limitation in host environments poses a significant challenge for fungal pathogens. Many host niches are glucose-poor, favouring microbes that utilise alternative carbon sources (Brink et al., 2021; Welte and Gould, 2017). To overcome this, pathogenic yeasts often secrete lipases to exploit host lipids as a carbon source (Stehr et al., 2003) (Figure 5). Notably, *C. neoformans* can harness lipids within macrophages by activating β-oxidation and upregulating genes involved in fatty acid metabolism (Fan et al., 2005; Hu et al., 2008). Under phosphorus starvation, fungi can adapt by remodelling their phospholipid metabolism, replacing phospholipids with a phosphorus-free betaine-lipid analogue to release phosphorus (Ray et al., 2019; Riekhof et al., 2014). Furthermore, lipid metabolism in fungi is intricately linked to iron homeostasis (Usmani et al., 2025). Since iron is a cofactor for enzymes in the ergosterol synthesis pathway, the ergosterol content in yeast cell membranes decreases during iron limitation, lowering the yeast’s iron requirement (Jordá et al., 2022; Prasad et al., 2006). It was also shown that *C. albicans* decreases the production of the non-essential lipid metabolite, PGE_2_, under iron-limited conditions (Mochochoko et al., 2021).

Lipids are not only required for fungal survival but are also mediators of virulence (Rella et al., 2016). In *C. albicans*, components such as phospholipids, ergosterol, and sphingolipids participate in yeast-to-hyphal transition and biofilm formation (Alim et al., 2018; Jain et al., 2022; Lattif et al., 2011; Liu et al., 2009). Membrane lipids may also partition proteins into ergosterol and sphingolipid-rich membrane microdomains, called lipid rafts (Eliaš et al., 2024). These lipids influence the biophysical properties of the lipid rafts, increasing the rigidity of the membrane domains in a liquid-ordered state (Alvarez et al., 2007). Lipid rafts were observed at the growing hyphal tip of *C. albicans* and is suggested to be required for hyphal formation. The interaction between these microdomains and certain proteins, especially GPI-anchored proteins, such as adhesins, may influence infection outcomes (Martin and Konopka, 2004). In *C. neoformans*, lipid rafts concentrated other virulence associated proteins, superoxide dismutase and phospholipase B1 (Siafakas et al., 2006). Interestingly, *C. neoformans* can also detect host phospholipids as a danger signal, leading to the formation of enlarged capsules (Chrisman et al., 2011). In addition, phospholipid homeostasis in *C. neoformans*, especially the presence of phosphatidylcholine, is crucial for capsule synthesis and cell wall composition (Lee et al., 2024). Recently, the ability of *C. albicans* to produce 13-hydroxyoctadecadienoic acid was reported. Importantly, this lipid molecule drives cytotoxicity within the host, independent of conventional virulence factors (Hitzler et al., 2025).

Several studies have highlighted the role of fungal membrane lipid modulation in resistance to antifungals, including azoles, polyenes and echinocandins (Ali et al., 2024; de Ghellinck et al., 2015; Gao et al., 2018; Huang et al., 2016; Kalra et al., 2024; Mukherjee et al., 2003; Pierce et al., 1978; Shor et al., 2016). Importantly, the composition of membrane lipids affects the localisation and function of drug efflux pumps (Mishra et al., 2008; Mukhopadhyay et al., 2002; Pasrija et al., 2008; Suchodolski et al., 2020). This highlights the complex interplay between membrane architecture, drug diffusion, and efflux pumps in modulating resistance to antifungal agents (Mukhopadhyay et al., 2002).

## 4 Insects as adaptive yeast vectors

Insects are the most successful group of animals on Earth as measured by species abundance, individual numbers, and habitat diversity (Blackwell, 2017). Notably, more than 6% of the yeast strains in the collection of the Westerdijk Fungal Biodiversity Institute originate from insect sources (Boekhout, 2005). Although a limited number of yeasts have been identified as primary or secondary symbionts of insects, the ability of yeasts to attract insects is likely a general ancestral trait that evolved before the emergence of angiosperms (Becher et al., 2018; Nwaefuna et al., 2023; Malassigné et al., 2021). Interestingly, filamentous fungi are more likely to be entomopathogenic, whereas little is known about entomopathogenic yeasts, suggesting that yeasts may have lost their ability to infect and cause disease within insects (de Bekker et al., 2021). Instead, yeast endosymbionts can modulate the immune response to maintain a stable mutualistic symbiotic relationship within their insect hosts (Malassigné et al., 2021; Pozo et al., 2020; Stefanini, 2018). This suggests that yeasts may have evolutionarily favoured mutualistic interactions over pathogenic ones as a strategy for ecological success, leveraging dispersal and resource exchange.

**Figure 6:**
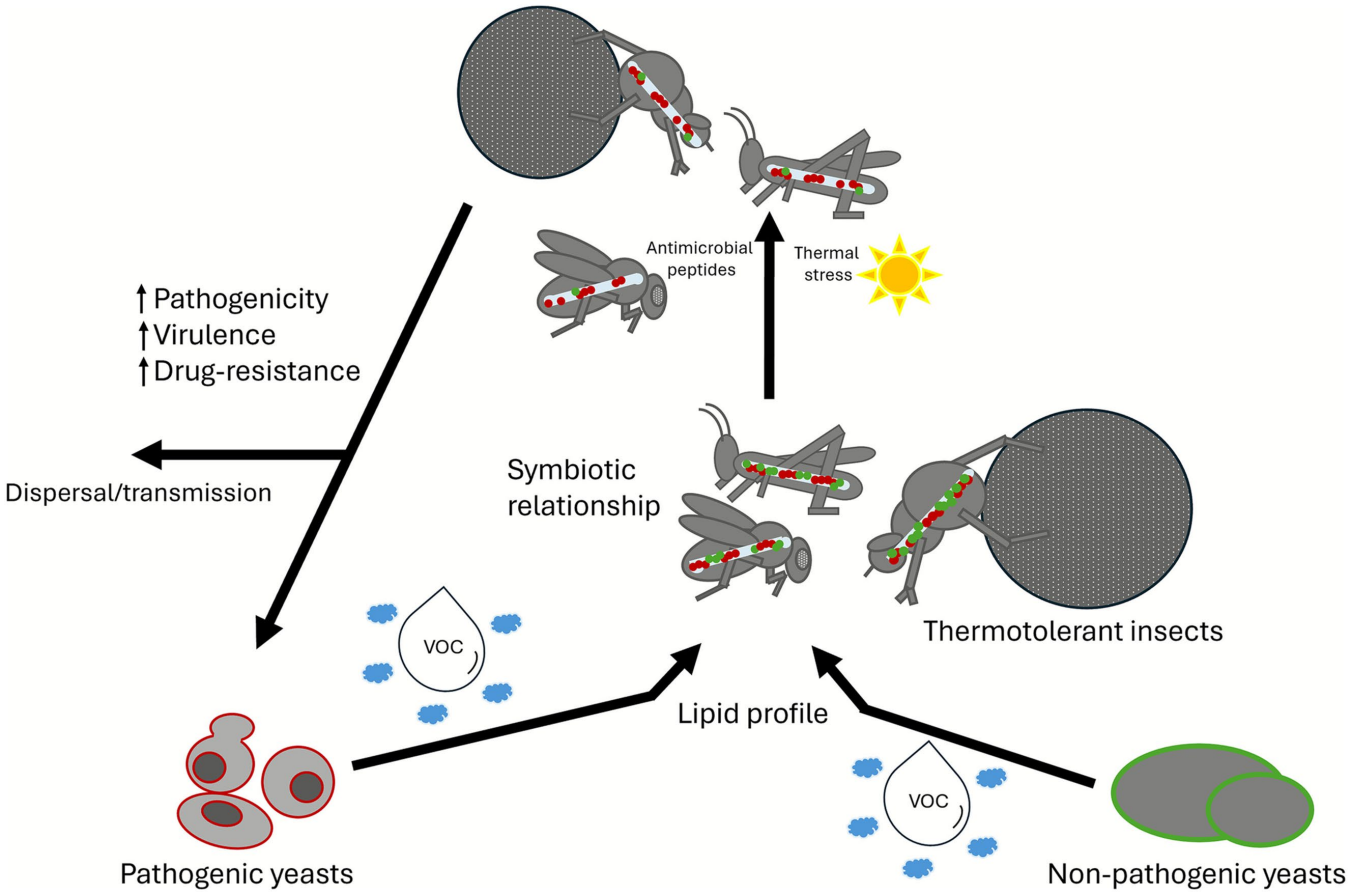
Selective pressure driving the evolution of pathogenic yeasts. The diagram illustrates how the lipid profile of pathogenic and non-pathogenic yeasts can attract thermotolerant insects through volatile organic compounds. Upon ingestion, the yeasts are exposed to selective pressures, including antimicrobial peptides and thermal stress, favoring the survival and selection of yeasts with enhanced pathogenic potential, which are then dispersed.

Mutualistic interactions between yeasts and insects are driven by complex nutritional dependencies and chemical signalling, with lipids playing a central role in shaping these associations. Insects are unable to synthesise sterols, certain vitamins and enzymes and many of these dietary requirements are provided by yeasts (Blackwell, 2017). Yeast can exploit this dependency to attract insects by generating a sensory impression of a desirable nutritional source in the insect’s nervous system (Stökl et al., 2010). For example, in ephemeral flower communities, *Drosophila* spp. are attracted to yeasts as a food source via volatile organic compounds (VOCs), produced during lipid metabolism, particularly from free fatty acids (Figure 6). Thus, VOC production, which may influence insect feeding preferences, is directly influenced by the yeast lipid profile (Bianchi et al., 2020). In return, the insect provides yeasts with a means of dispersal and a protected environment for growth and reproduction (Stefanini, 2018). Yeast lipid composition is shaped by factors like carbon source and growth medium, and further modified by interactions with insects (Bianchi et al., 2020). Many insects show preferences for specific lipids, indicating that the ability to detect lipids may be a widespread and ecologically relevant trait among insect taxa (Blaul and Ruther, 2011; Brankatschk et al., 2018; Ugine et al., 2022). Therefore, it is possible that yeast lipid composition plays a key role in mediating the yeast-insect association and the use of insects as dispersal vectors (Figure 6). Consequently, insects’ reliance on lipid-derived signals and imposition of lipid-targeted stresses likely exert selective pressure important for shaping the yeast lipidomes.

Thermal biology plays a critical role in shaping the interactions between insects and their microbial symbionts or pathogens. Insects employ various strategies for regulating body temperature, including behavioural thermoregulation (Lahondère, 2023; Willott, 1997). Thermotolerant insects, such as locusts and grasshoppers, actively maintain optimal body temperatures between 38 °C and 40 °C, which enhances their immune response and inhibits the growth of pathogens, thereby defending against infection (Thomas and Blanford, 2003). Similarly, many ectothermic insects like dung beetles require a thoracic temperature of at least 25 °C or 30 °C to sustain flight, with the maximum temperature tolerated during flight being 42 °C, near the heat shock limit (Verdú et al., 2006). In grasshoppers and locusts, the virulence of fungal pathogens and the resulting host mortality rates are highly dependent on temperature, with even a slight temperature change transforming an entomopathogen from highly virulent to nearly harmless (Robert and Casadevall, 2009; Thomas and Blanford, 2003). These thermal constraints exert a strong selective pressure, favouring yeasts that are thermotolerant and able to function at temperatures optimal for the host’s physiology and behaviour.

## 5 Insects as ecological bridges in the evolution of pathogenic yeasts

It is evident that there is a close association between yeasts and insects, with insects acting as potential vectors, facilitating the spread of yeasts to new environments. Emerging evidence also suggests that insects may enhance yeast persistence in the environment and their adaptation to multiple stressors, such as insect-produced antimicrobial compounds, including lipids (free fatty acids, fatty alcohols and fatty acid-derived eicosanoids), lipid-associated proteins, and antimicrobial peptides (Wrońska et al., 2023). Many yeasts (including clinically relevant pathogens) isolated from dung beetles in Botswana were tolerant of stresses that they may experience in the mammalian host (Nwaefuna et al., 2023). In addition, recent work indicated that the brown locust from the Karoo region of South Africa harbours the pathogenic yeasts, *Candida orthopsilosis* and fluconazole-resistant *C. auris* (Ogundeji et al., 2025). Interestingly, insect antifungal peptides act upon different yeasts depending on the composition of their lipid membranes, favouring the survival of fluconazole-resistant isolates (Kodedová et al., 2019). Furthermore, some studies have linked the evolution of pathogenic yeast lineages, such as *Cryptococcus*, to African tree-infesting bark beetles, suggesting a potential association between these pathogens and insect vectors (Coelho et al., 2025). This suggests that insect-derived stress may influence yeast lipid plasticity and subsequent antifungal stress resistance (Figure 6). Thus, exposure to insect hosts may exert selective pressure on yeast lipidomes, driving the emergence of stress-tolerant, and potentially more virulent strains with more adaptable lipid metabolisms.

## 6 Potential One Health implications

Insects are increasingly recognised as keystone members of food webs, with important roles in cycling of nutrients through different trophic levels, often in concert with microbes (Yang and Gratton, 2014). In addition, iInsects, including locusts, are not only regularly consumed by humans, but are increasingly used as a highly nutritious, environmentally friendly, and cost-effective feed for livestock, pets and in aquaculture (Barroso et al., 2014; Zou et al., 2024; Gautam et al., 2025). From a One Health perspective this establishes ecological interfaces where yeasts can adapt and move across different hosts. Within these interfaces, insects impose distinctive **s**elective pressures that favour lipid metabolic plasticity in associated yeasts. As discussed, such plasticity, involving dynamic remodelling of fatty acids and sterols, is a known route to thermotolerance and stress survival, and may thereby potentiate pathogenic emergence. Consistent with this, the emerging human pathogen *Candida palmioleophila*, recently isolated from thermotolerant Sciaenidae fish in aquaculture (Oliveira et al., 2025), exhibits both growth at elevated temperatures (up to 42 °C) and lipolytic capacity, including the ability to utilize oils as sole carbon sources and express lipase-like proteins under lipid-rich conditions (Nakase et al., 1988; Rincón et al., 2018). These traits underscore how insect-enabled food and feed webs can select for lipid-flexible, heat-adapted yeast phenotypes with clinical relevance. Understanding these mechanisms is therefore critical for anticipating and mitigating yeast pathogenic emergence across human–animal–environment interfaces.

## 7 Future directions

To investigate the hypothesis that lipid metabolism plasticity, selected for by thermotolerant insects, enables yeasts to survive across diverse hosts, including mammals as potential pathogens, it is essential to conduct *in vivo* adaptation and evolution experiments using insect models capable of tolerating a wide temperature range. *Galleria mellonella*, which can survive temperatures from 20 °C to 42 °C (Firacative et al., 2020), serves as a suitable model for such studies (Ali et al., 2020). Environmental yeast strains adapted by passage through the digestive tract of such model insects, should be assessed for changes in lipid content, lipidome composition, and virulence in mammalian hosts. In addition, the ability of such passaged yeasts to be transmitted from insects to mammals (either directly via feed or indirectly through their persistence in the environment) should be assessed. Aspects that could be included in such studies are potential trade-offs or constraints, including fitness costs in non-insect environments, associated with selected lipid metabolic plasticity. Complementing these experiments, future research should include comprehensive field studies to isolate and identify thermotolerant insect-associated yeasts from diverse environments. Characterising the lipid profiles and virulence traits of these isolates may provide critical insights into their pathogenic potential. A multi-level approach—spanning gene/protein, genome, guild, and community levels—should be employed to explore the functional traits, their plasticity and environmental interactions. Additionally, investigating correlations between passaged yeasts, lipid profiles, virulence traits, and thermotolerant insect hosts will illuminate the mechanisms by which insects select for specific yeast lipid profiles. This integrated research strategy will ultimately deepen our understanding of how insect hosts influence yeast lipid metabolism and virulence, revealing the complex dynamics underpinning host-pathogen evolution.

## Data Availability

The original contributions presented in the study are included in the article/supplementary material, further inquiries can be directed to the corresponding author.
